# Mobile-CRISPRi protocol optimization for *Vibrionaceae*

**DOI:** 10.1128/mra.00040-24

**Published:** 2024-07-05

**Authors:** Logan J. Geyman, Madeline P. Tanner, Natalia Rosario-Meléndez, Jason M. Peters, Mark J. Mandel, Julia C. van Kessel

**Affiliations:** 1 Department of Biology, Indiana University, Bloomington, Indiana, USA; 2 Department of Medical Microbiology and Immunology, University of Wisconsin-Madison, Madison, Wisconsin, USA; 3 Microbiology Doctoral Training Program, University of Wisconsin-Madison, Madison, Wisconsin, USA; 4 Pharmaceutical Sciences Division, School of Pharmacy, University of Wisconsin-Madison, Madison, Wisconsin, USA; Indiana University, Bloomington, Indiana, USA

**Keywords:** CRISPR, CRISPRi, *Vibrio*, *Vibrio fischeri*, *Vibrio parahaemolyticus*, *Vibrio vulnificus*, *Vibrio campbellii*, *Vibrio cholerae*, regulation of gene expression

## Abstract

Mobile clustered regularly interspaced palindromic repeats interference (Mobile-CRISPRi) is an established method for bacterial gene expression knockdown. The deactivated Cas9 protein and guide RNA are isopropyl β-D-1-thiogalactopyranoside inducible, and all components are integrated into the chromosome via Tn*7* transposition. Here, we optimized methods specific for applying Mobile-CRISPRi in multiple *Vibrio* species.

## ANNOUNCEMENT

*Vibrio* bacteria are important model organisms for diverse fields of research. Their use in research has predicated the demand for increasingly creative genetic tools. While many *Vibrio* species are naturally competent (1), other models remain more genetically recalcitrant. Mobile clustered regularly interspaced palindromic repeats interference (Mobile-CRISPRi) was initially demonstrated in non-model *Vibrio caseii*, and its potential made it a target for further optimization in model Vibrio species (2, 3). Herein, we specify the optimized workflow for Mobile-CRISPRi in *Vibrio*.

**Fig 1:**
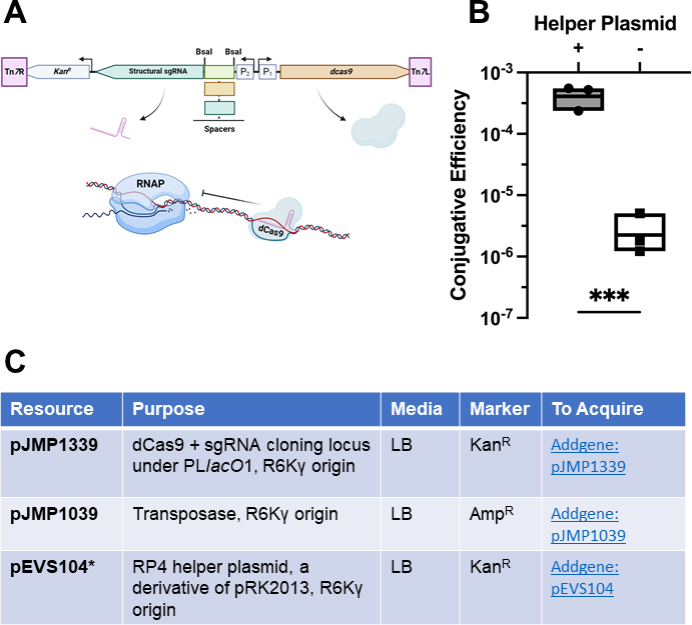
(**A**) Schematic for Mobile-CRISPRi. P1 and P2 are both PL*lacO*1 (6). (**B**) Conjugative efficiency of plasmid pLG2 targeting *luxR* in *V. campbellii* with and without RP4 helper plasmid pRK600 (3). The line in each box represents the group mean. Each point is a biological replicate that is the average of two technical replicates. Statistical analysis performed using GraphPad Prism: unpaired *t*-test on log-transformed data. ***P = 0.0005. (**C**) Table outlining the conjugation resources available for this method. *pEVS104 was not the helper used in panel **B**.

Mobile-CRISPRi utilizes inducible expression of deactivated Cas9 and a single-guide RNA (sgRNA), both expressed through isopropyl β-D-1-thiogalactopyranoside-dependent induction, to facilitate conditional knockdown of target genes. This system is stably incorporated endogenously into the target organism via Tn*7* transposition (Fig. 1A). This is typically done using a tri-parental conjugation, with diaminopimelic acid (DAP) counter-selection of the plasmid donors and antibiotic selection for successful target organism integration. When optimizing this system for use in *Vibrio*, we observed a limiting deficiency in successful conjugates, typically yielding exconjugants in *Vibrio fischeri* but not the other species tested. To address this issue, we deployed an *Escherichia coli* strain containing an RP4 helper plasmid, pRK600, a pRK2013 derivative (4, 5). We found that quadraparental mating improved conjugative efficiency by ~100-fold in *Vibrio campbellii* (Fig. 1B). As a result, we have optimized this protocol for non-*fischeri Vibrio* as follows:

1. Acquire the necessary vectors from Addgene (see Fig. 1C).
2. Clone desired sgRNA into pJMP1339 following the protocol from Banta et al. (6).
  - Note that the R6Kγ origins require the use of cloning strains that possess *pir* (e.g., λ *pir*).
3. Grow up overnight cultures of *E. coli* strains containing the following plasmids in appropriate antibiotic-containing Lysogeny Broth (LB) media:
  - pJMP1039 (transposase).
  - Modified pJMP1339 with the cloned sgRNA.
  - Target bacterial strain.
  - pEVS104 helper plasmid.
    - Note: While plasmid pRK600 is not available in public repositories, pEVS104 is an alternate RP4 helper plasmid modified for use in *Vibrio* that is publicly available through Addgene (Fig. 1C) (5).
4. Spin down 1 mL of each culture in a tabletop centrifuge for 5 minutes at 8,000 × *g* and resuspend in antibiotic-free LB medium.
5. Following this, co-spot 10 µL of each culture onto an LB plate. Repeat this three times to create four co-spots on the same LB plate. Incubate overnight at 30°C with the lid up.
6. The next day, resuspend all four of the co-spots in the same 1 mL of LB-Marine (LM): LB supplemented with 10 g of NaCl per liter. Streak the co-spot mix for single colonies on LM plates supplemented with polymyxin B to counter-select against *E. coli* (see note below) and 100-µg/mL kanamycin to select for successful Mobile-CRISPRi plasmid integration. Incubate plates at 30°C overnight.
  - For *Vibrio* strains that are not naturally polymyxin B resistant, DAP auxotrophic *E. coli* should be used and the co-spots streaked on LM without DAP to counter-select against. *E. coli*.
  - We recommend plating for viability on both the plates listed above and a plate group without kanamycin to calculate conjugative efficiency out of the total viable cells. In Fig. 1B, these values were calculated by dividing the CFUs on the polymyxin B + kanamycin plates by those present on plates with polymyxin alone.
7. Upon the growth of colonies, test a small subset of exconjugants (two to three) for integration via colony PCR using a primer that sits in the *glmS* locus and one that sits within the Mobile-CRISPRi near the Tn*7*-R site.

The same concept can be applied in *V. fischeri* using conjugation and media conditions described by Stabb and Ruby (5). Use of the DAP counter-selection against the *E. coli* donor is especially useful in *V. fischeri*.

Further details of how this toolkit functions experimentally in *Vibrio* are available here (3). The use of the RP4 helper plasmid greatly expanded the efficiency of this system (Fig. 1B). This modification has made the use of Mobile-CRISPRi a reliable, robust toolkit for exploring *Vibrio* genetics (3).
